# A review of three decades of use of the cattle brucellosis rough vaccine *Brucella abortus* RB51: myths and facts

**DOI:** 10.1186/s12917-023-03773-3

**Published:** 2023-10-18

**Authors:** J. M. Blasco, E. Moreno, P. M. Muñoz, R. Conde-Álvarez, I. Moriyón

**Affiliations:** 1 Instituto Agroalimentario de Aragón-IA2 (CITA-Universidad de Zaragoza), Zaragoza, España; 2 Programa de Investigación en Enfermedades Tropicales, Escuela de Medicina Veterinaria, Universidad Nacional, Heredia, Costa Rica; 3 Departamento de Ciencia Animal, Centro de Investigación y Tecnología Agroalimentaria de Aragón (CITA), Zaragoza, España; 4 Instituto de Investigación Sanitaria de Navarra and Departamento de Microbiología y Parasitología, Universidad de Navarra, Pamplona, Spain

**Keywords:** Brucellosis, *B. abortus*, *B. melitensis*, Cattle, Vaccine, Control, Eradication, Serologial diagnosis, RB51, DIVA

## Abstract

Cattle brucellosis is a severe zoonosis of worldwide distribution caused by *Brucella abortus* and *B. melitensis*. In some countries with appropriate infrastructure, animal tagging and movement control, eradication was possible through efficient diagnosis and vaccination with *B. abortus* S19, usually combined with test-and-slaughter (T/S). Although S19 elicits anti-smooth lipopolysaccharide antibodies that may interfere in the differentiation of infected and vaccinated animals (DIVA), this issue is minimized using appropriate S19 vaccination protocols and irrelevant when high-prevalence makes mass vaccination necessary or when eradication requisites are not met. However, S19 has been broadly replaced by vaccine RB51 (a rifampin-resistant rough mutant) as it is widely accepted that is DIVA, safe and as protective as S19. These RB51 properties are critically reviewed here using the evidence accumulated in the last 35 years. Controlled experiments and field evidence shows that RB51 interferes in immunosorbent assays (iELISA, cELISA and others) and in complement fixation, issues accentuated by revaccinating animals previously immunized with RB51 or S19. Moreover, contacts with virulent brucellae elicit anti-smooth lipopolysaccharide antibodies in RB51 vaccinated animals. Thus, accepting that RB51 is truly DIVA results in extended diagnostic confusions and, when combined with T/S, unnecessary over-culling. Studies supporting the safety of RB51 are flawed and, on the contrary, there is solid evidence that RB51 is excreted in milk and abortifacient in pregnant animals, thus being released in abortions and vaginal fluids. These problems are accentuated by the RB51 virulence in humans, lack diagnostic serological tests detecting these infections and RB51 rifampicin resistance. In controlled experiments, protection by RB51 compares unfavorably with S19 and lasts less than four years with no evidence that RB51-revaccination bolsters immunity, and field studies reporting its usefulness are flawed. There is no evidence that RB51 protects cattle against *B. melitensis,* infection common when raised together with small ruminants. Finally, data acumulated during cattle brucellosis eradication in Spain shows that S19-T/S is far more efficacious than RB51-T/S, which does not differ from T/S alone. We conclude that the assumption that RB51 is DIVA, safe, and efficaceous results from the uncritical repetition of imperfectly examined evidence, and advise against its use.

## Background

The brucellae are gram-negative pathogens related to free-living bacteria of the genus *Ochrobactrum* [1]. Brucellosis, the disease they cause, is a worldwide extended zoonosis severely affecting many domestic and wild animals*.* Bovine brucellosis is primarily caused by *Brucella abortus*, a species that can infect water buffaloes, yaks, camels, and, less frequently, dogs, small ruminants, horses, pigs, reindeer and some wild animals [2, 3]. Moreover, contrary to some assumptions [4], there is conclusive evidence that cattle can be infected by *B. melitensis,* the most typical cause of sheep and goat brucellosis, and that this is not a spill-over infection spontaneously clearing once the infected small ruminants are removed [5–15]. Also, *B. suis*, the typical agent of swine brucellosis, can very rarely infect cattle [16–18]. In humans, these three *Brucella* species produce a severe and debilitating disease requiring prolonged combined antibiotherapy that, if untreated, can produce disabling sequelae and death [19, 20]. Contact with animals and their products and ingesting unpasteurized dairy products are significant sources of human brucellosis. Consequently, the control and eventual eradication of brucellosis in domestic ruminants improve animal production and minimize its zoonotic impact [21].

Controlling (i.e., lowering the prevalence to reduce its spread and socioeconomic impact) and eradicating brucellosis in domestic ruminants is far from easy. One immediate approach is identifying (commonly by serological testing) and culling infected animals. However, these "test-and-slaughter" (T/S) programs are costly and only under very favorable conditions achieve the control pressure necessary to prevent the spread of brucellosis when herd prevalence is high. This strategy explains why, without vaccines, T/S programs have succeeded only in some Scandinavian areas, all with small herds, tight control of animal movements, adequate veterinary infrastructure, and suitable budget [22, 23]. Although with appropriate infrastructural and budgetary conditions, for the remaining handful of countries that eliminated bovine brucellosis, the attenuated live smooth (S) *B. abortus* S19 vaccine was key for reducing the herd prevalence in most epidemiological and breeding systems before achieving eradiation through just T/S. Canada, the U.S.A., New Zealand, Australia, and several European Union (E.U.) countries, all with significant numbers of cattle, systematically used S19 as a previous step to implement eradication by T/S strategies. Applied initially as 10^11^ colony forming units (CFU) subcutaneous dose, mainly in young replacement heifers, the S19 abortifacient effect, milk excretion, and long-lasting antibody response against the diagnostically relevant O-polysaccharide antigen (O-PS) of the lipopolysaccharide (LPS) [24] were minimized four decades ago by applying a single reduced dose (5 × 10^9^ CFU) conjunctively. This vaccination protocol does not require a booster or reduces the protection conferred by the subcutaneous vaccination. Moreover, it minimizes abortions and bacterial milk secretion and is suited for most epidemiological settings, including mass vaccination [25]. Hence, S19 is the gold standard against other vaccines should be compared.

Bovine brucellosis is endemic in Latin America, Africa, and Asia, including fast-growing economies that, like China, should not be affected by infrastructural and budgetary shortcomings. The fact is that where control was or is being unsuccessfully attempted, even more than in economic and veterinary service deficiencies, the failure is rooted in extended misunderstandings of control strategies, diagnostic tools, and vaccines. We have analyzed these issues before, mainly concerning control strategies and diagnostic tools [25–28]. Based on the experimental results and field experience gained in the last decades, we complement these analyses by updating the information on *B. abortus* RB51, a widely used bovine brucellosis vaccine thought to have properties that advise its use over that of S19.

### *B. abortus* RB51 vaccine: a historical perspective

In countries that achieved eradication, vaccination with S19 was generally interrupted when herd seroprevalence was very low. This strategy facilitated unambiguously identifying the few infected individuals by testing for antibodies reacting in smooth (S) LPS (S-LPS) tests, which offer the highest diagnostic sensitivity [27]. While this policy was successfully applied in these wealthy countries, a widespread mistake in latitudes with less favorable epidemiological and economic conditions has been an ill-timed interruption of S19 vaccination fueled by a misunderstanding of this strategy [28]. Certainly, discontinuing vaccination would not be necessary with a vaccine enabling the differentiation of infected and vaccinated animals (DIVA). Thus, as countries like the U.S.A., Canada, and Australia approached eradication in the last decades of the past century, there was a renewed interest in the so-called *Brucella* rough (R) vaccines. These live vaccines use R mutants that lack the O-PS and keep the internal (core and lipid A, essential for viability) LPS sections. Since the O-PS epitopes are those relevant in S-LPS tests [24], an assumed property of R vaccines is that they should be DIVA when combined with tests that detect anti-O-PS antibodies [29, 30].

The only R vaccine commercialized and extensively used is *B. abortus* RB51, an R mutant derived from *B. abortus* 2308, an S virulent challenge strain used in vaccine experiments. RB51 carries a mutation in *wboA* (coding for an O-PS glycosyltransferase), possibly in *capD* (also known as *wbkD*, the putative epimerase/dehydratase proposed to be involved in the initial steps of O-PS synthesis [31], in *eipA* (cell envelope homeostasis) and *narJ* (intracellular survival) [32]. Still, this strain produces small quantities of O-PS-like molecules [33], in all likelihood on account of the remaining intact *wbk* O-PS genes [31]. Clearly, this set of mutations explains the over-attenuation and reduced vaccine efficacy in mice of RB51 when compared to O-PS glycosyltransferase single mutants or S19 [30, 34]. Obtained by repeated passages on rifampin-containing agar, RB51 also carries a mutation in *rpoB* that accounts for its resistance to this antibiotic, an unfortunate trait because rifampin is used for human brucellosis treatment [35]. This altered RpoB may also contribute to its over-attenuation by hampering protein synthesis fitness [36]. Upon isolation, RB51 can be identified by PCR targeted to one of the genetic defects, as in the OIE-recommended Bruceladder [2, 37]. However, in contrast to wild-type *B. abortus*, the RB51 defects make it highly sensitive to the inhibitory agents in the routinely used Farrell’s *Brucella* selective media, and also in *Brucella* Ewalt’s and Kuzdas and Morse and modifed Thayer-Martin media [38]. Thus, when RB51 is involved, efficient bacteriological investigation of animal samples requires selective media allowing the growth of both field strains and RB51, such as CITA agar or a combination of both Farrell’s and CITA [39]. These precautions are of paramount importance to evaluate RB51 safety and efficacy experiments.

In 1990, just after bovine brucellosis was practically eradicated in the U.S.A., RB51 was registered and then introduced in 1996 based on initial reports of its safety, efficacy, and DIVA properties [29, 30, 40]. Although in 2003 the company marketing RB51 claimed that “*For the past seven years, the vaccine … has served as a significant factor in the government’s 50-plus year effort to eradicate brucellosis from the United States*” (sic.) [41], by 1996 the disease had been practically eradicated in the U.S.A. without making use of RB51 [42]. Not unexpectedly, the experiments supporting those claims were questioned afterwards [30]. Nevertheless, RB51 was soon introduced in many countries following the broadcasting of apparently promising results [43, 44] and sustained marketing campaigns in countries where a deficient application of S19 had raised doubts about the usefulness of this vaccine [28], and S19 abandoned or even banned. These low and middle-income countries included some African and all Latin American nations that had never systematically applied T/S after vaccination. It also included European countries such as Portugal, Italy, Greece, and Spain experiencing delays in expensive E.U.-sponsored eradication programs.

### RB51 vaccination protocols

After nearly three decades, and in contrast with S19 [25], there is no standard operating procedure for RB51 administration, and different and contradictory methods are used. Upon its introduction in the U.S.A., a single subcutaneous dose of 1.0–3.4 × 10^10^ CFU (the so-called RB51 “full dose”) was applied to calves and heifers of 4–12 months of age, and a reduced dose of 1.0–3.0 × 10^9^ CFU to cows older than 12 months [45]. However, current guidelines by the same producer and the USDA limit its use to 4 to 12 months old heifers in the U.S.A. [41, 46] and allow the use of RB51 in older animals only if proof of previous vaccination is not available [47]. Shortly after its introduction in Mexico, heifers were vaccinated with the full dose at 5 months of age, revaccinated twice 6 and 12 months later, and additional revaccination of adults with a reduced dose (1.0–3.0 × 10^9^ CFU) was recommended in case of outbreaks [48]. Some authors revaccinated adult cattle (including pregnant ones) previously vaccinated with S19 with a reduced dose of RB51 [49, 50], and this practice has been adopted in some countries. In Brazil, the current control program is based on the vaccination of 3–8 months old calves with S19, RB51 vaccination of older females that were not S19 vaccinated, and, in outbreaks, mass-vaccination with RB51 of previously vaccinated herds [51, 52]. Strikingly, in different countries, the instructions of the dealers vary. For instance, in South Africa, the widely marketed Bovilis ® RB51 [53] instructs to vaccinate with full doses 4–10 months old calves, revaccinate 12–16 months old heifers and vaccinate non-pregnant adult cows, with yearly boosters “*if desired*” (sic.). Not infrequently, the benefits of revaccination are assumed and manufacturer instructions go unheeded: in some regions of Spain, heifers and adult cows were vaccinated with the full dose, revaccinated 6 months later, and then up to four more times at yearly intervals with the same full dose [54].

### RB51 as a DIVA vaccine

It has been reported repeatedly that RB51 does not elicit anti-O-PS antibodies regardless of the age, physiological condition, dose, and frequency of revaccination [55–57]; for earlier literature, see [30, 58]. These reports, that were based on experiments conducted in *Brucella*-free environments using agglutination tests, have led to the conviction that this property allows a straightforward interpretation of serological tests in all epidemiological circumstances [59, 60]. According to this idea, cattle vaccinated and/or revaccinated with RB51 (including those previously immunized with S19) that are not subsequently infected can be discriminated from the infected ones by a negative result in S-LPS serological tests even in infected environments, the circumstance that justifies vaccination. However, it is evident that no vaccine can impede exposure to the pathogen in endemic areas and immunity activation by this challenge.

**Fig. 1:**
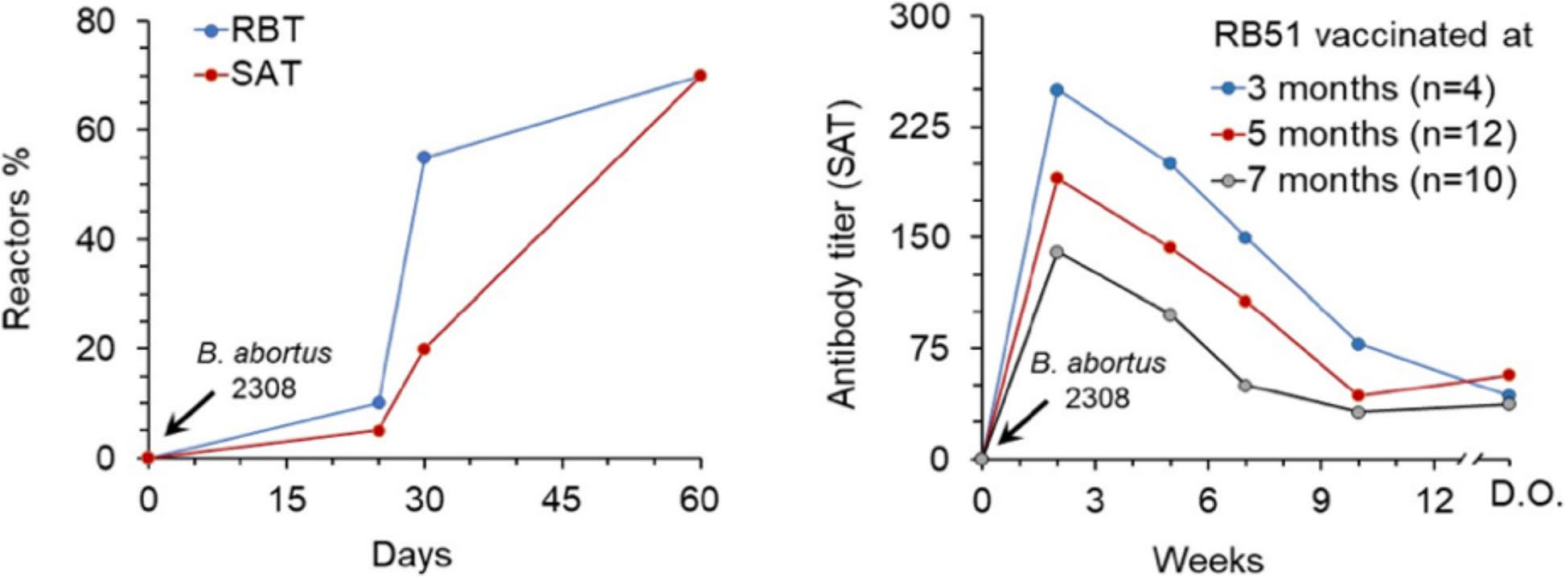
Agglutinating antibodies developed by RB51 vaccinated cattle after challenge with virulent *B. abortus* 2308. Left panel, evolution of the percentage of Standard agglutination (SAT) and Rose Bengal (RBT) reactor cows vaccinated at 24 months of age after challenge (arrow) at 6–7 months of pregnancy (data from [61]). Right panel, evolution of SAT titers in RB51 vaccinated heifers at the indicated age after challenge (arrow) (D.O. delivery outcome, either parturition or abortion) (adapted from [62])

As expected, the first experiments conducted under controlled conditions showed that challenge with virulent S brucellae generates anti-OPS antibodies in RB51 vaccinated animals. Figure 1 summarizes representative experiments conducted in RB51 vaccinated cows that were challenged with *B. abortus* 2308 and then examined with the rose bengal (RBT) and standard agglutination (SAT) tests that detect anti-O-PS antibodies [24]. In one experiment (left panel; [61]), nearly 60% of pregnant cows were RBT positive by the first month after the challenge, and about 70% were positive in both RBT and SAT two months later. The other experiment (right panel; [62]) shows that all calves became SAT-positive within 3 weeks. Similar results can be found in other works that show no significant differences in SAT titers between RB51 vaccinated and unvaccinated controls after the challenge [63–65]. Consistent with the controlled experiments, cattle vaccinated with RB51 that are in infected enviroments also develop anti-O-PS antibodies. In one study, 6 out of 35 RB51 vaccinated and revaccinated cattle from a brucellosis-free herd became positive in the RBT-like Card Test after being moved into an infected herd [66]. Indeed, this is true of challenges conducted with other S brucellae. Upon challenge with virulent *B. suis*, the buffered acid plate agglutination test (BAPAT) and the fluorescence polarization assay (FPA) yielded similar results in RB51 vaccinated and unvaccinated cattle, with both tests peaking 4–8 weeks after exposure and remaining positive at least up to week 12 [67].

In cattle previously vaccinated with S19, anamnestic responses elicited by the O-PS-like molecules in RB51 [33] can also cause interferences even in the absence of contact with wild-type brucellae. It has been reported that 2 out of 38 and 7 out of 34 cows seronegative after S19 vaccination seroconverted in the Card and BAPAT tests after revaccination with reduced doses of RB51 [68]. While the problem was solved (but not clearly, see below) by the subsequent use of the complement fixation test (CFT), this protocol of vaccination/revaccination leads to a combined RBT-CFT diagnostic strategy that is considered a main inconvenience of S19 that RB51 circumvents. Indeed, the animals in this experiment were not exposed to virulent brucellae, which would have further complicated the interpretation of serological tests, as discussed above.

**Fig. 2:**
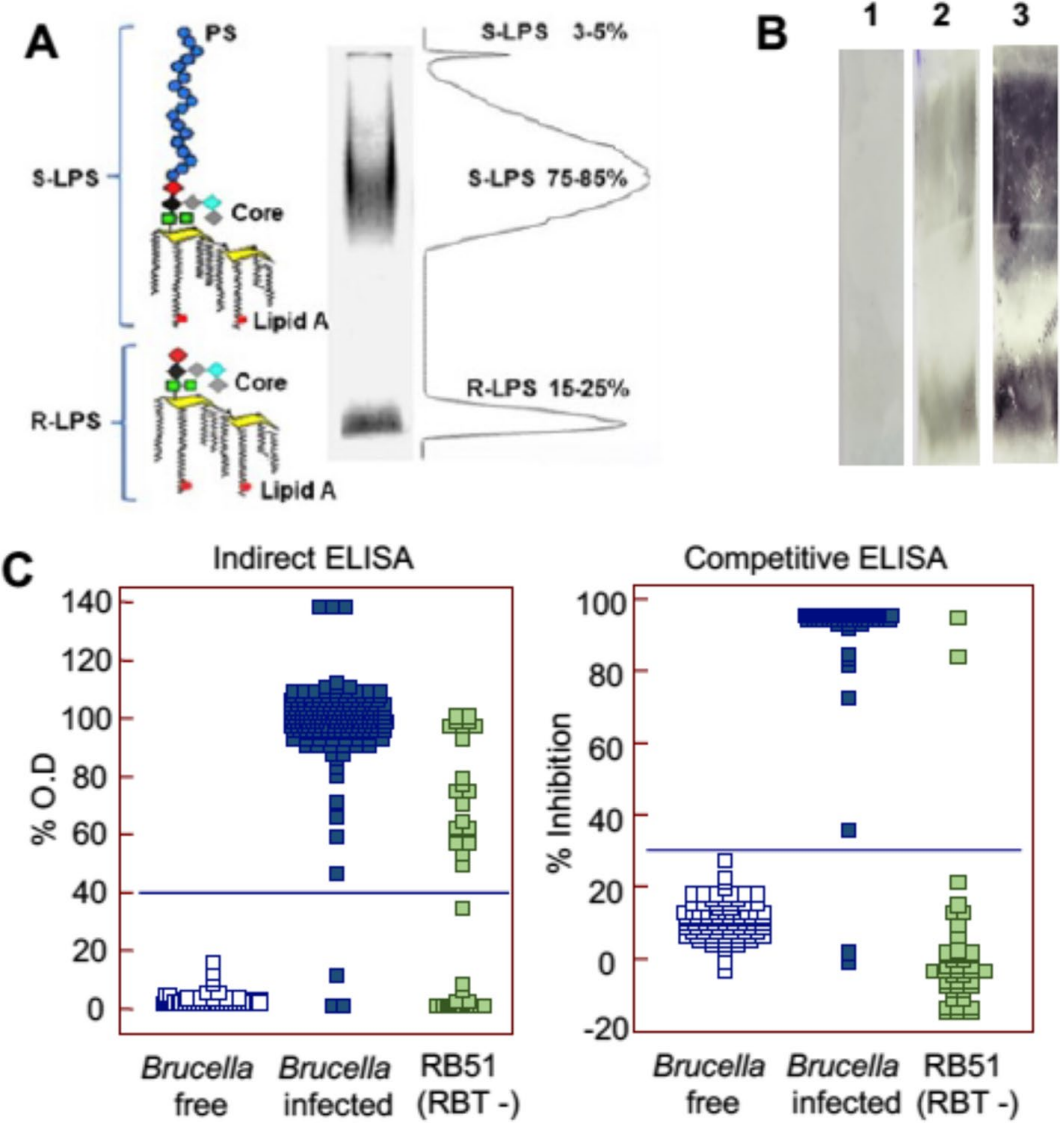
*Brucella* LPS epitopes and antibody response in RB51 vaccinated cattle. **A** Schematic representation of S- and R-LPSs and SDS-PAGE of silver-stained protein-free S-LPS [70] from *B. abortus* 2308 revealing by densitometry the proportion range of R- and S-LPSs (adapted from [69]). **B** Western-blot performed with protein-free S-LPS and sera from cattle of brucellosis-free herds. (1) Unvaccinated heifer (negative control); (2) positive reaction with the serum of a RB51 vaccinated heifer that aborted after vaccination and from which RB51 was isolated (this serum was taken 12 months after vaccination); (3) strong positive control with the serum of a rabbit hyperimmunized with acetone-killed RB51 cells. **C** Serological responses of RB51 vaccinated cattle in iELISA (INGEZIM Brucella Bovina 2.0 -Gold Standard Diagnostics-, left) and cELISA (Svanovir Brucella-Ab C-ELISA -Svanova-, right) commercial tests for the diagnosis of bovine brucellosis performed following the manufacturing instructions. The cut-off (line in blue) resulting in maximal diagnostic sensitivity and 100% Diagnostic Specificity was established in both tests with a representative collection of gold standard sera taken from *B. abortus* culture positive and brucellosis free cows. Sera from RB51 vaccinated RBT-negative cows (green squares) were obtained from 5 to 8-month-old brucellosis-free heifers vaccinated with the full dose (1–3.4 × 10^10^ CFU) of RB51 (CZ Vaccines. Porriño. Spain), bled between 9 and 18 months after vaccination and maintained in a brucellosis-free environment

In addition to the interference of the anti-S-LPS response in endemic areas and revaccination of S19 immunized heifers, immunosorbent assays such as indirect ELISAs (iELISA), competitive ELISAs (cELISA), and lateral flow immunochromatography [LFiC]) are further affected by other issues. Figure 2, panel A, illustrates that S-LPS preparations obtained from S brucellae do not have a homogeneous molecular weight. Owing to the mechanism of S-LPS synthesis, LPS extracts from S brucellae show O-PS heterogeneity and the presence of R-LPS (i.e., lipid A-core molecules on which the O-PS has not been assembled, about 10% of the total according to [69]). Whereas in the surface of intact unheated bacteria (see below), the S-LPS hides most lipid A-core epitopes, these become exposed upon adsorption of inactivated cells or S-LPS extracts to the matrixes used in ELISAs or LFiC [24]. Using sera from animals vaccinated with RB51, this effect can be demonstrated unequivocally by Western blot performed with extensively purified S-LPS [70] to avoid detecting antibodies of other specificities elicited by RB51 [71, 72] (Fig. 2, panel B). Indeed, antibodies to core-lipid A are elicited by both R and S brucellae, which, together with the exposure of core-lipid A determinants, make the respective responses overlap in binding assays [73, 74]. This phenomenon is noted in iELISA performed with S-LPS, even when optimized for diagnosing infections by wildtype *B. abortus* (or other S brucellae). In one study, 8% and 24% of adult cows (*n* = 25), vaccinated and revaccinated with reduced doses of RB51, respectively, became positive in an S-LPS iELISA adjusted to discriminate *B. abortus* infections of cattle, with responses peaking 30 days after vaccination and lasting for up to 6 months in the revaccinated group [75]. In another study [76], brucellosis-free heifers vaccinated with a full-dose of RB51 and maintained in a brucellosis-free environment were tested 9–18 weeks after vaccination. Of 22 animals, 50% resulted positive in LFiC and 77% in an S-LPS iELISA, both tests optimized for *B. abortus* diagnosis. As illustrated in Fig. 2, panel C, these issues are not solved by cELISA. Furthermore, re-adjusting the cut-off of either iELISAs or cELISAs to discriminate RB51 vaccinated animals would result in a total loss of diagnostic sensitivity in detecting infected animals. All these data illustrate that RB51 causes severe interference in immunosorbent assays optimized to detect truly infected cattle, even in brucellosis-free contexts. This phenomenon is an intrinsic limitation of the R vaccination approach, as demonstrated by the observations made with genetically defined R *B. melitensis* mutants in controlled experiments [77].

A similar problem affects FPA and other binding assays that use S-LPS hydrolytic polysaccharides because hydrolysis removes the lipid A but not the core epitopes that then become exposed in the solution. FPA has been shown to detect antibodies triggered by R brucellae [24] and, although a study reported 100% specific in discriminating RB51 vaccinated and revaccinated cattle, the positive controls were cattle experimentally infected with *B. abortus* [78] and the background threshold reactivity was established with only a few sera [5, one in the FPA kit, and 4 from herds, plus 10 negative controls]. This limitation and the fact that experimentally infected animals are not representative of the naturally infected ones [27, 79, 80] show that such FPA perfect specificity is an unsupported conclusion.

The interference caused by antibodies to core-lipid A epitopes also affects the CFT, which is puzzling because the antigen is a suspension of whole S bacteria. Revaccination with RB51, technical aspects of the antigen preparation, and differences in the antibody effects detected in different whole S bacteria tests can account for this, as discussed next.

On December 6th, 2009, 40 Simmental (Fleckvieh) adult cows from an unvaccinated brucellosis-free herd in Spain Segovia Province were vaccinated with a full dose of RB51 following the manufacturer’s instructions (CZ Vaccines) and kept isolated in a closed farm without contact with other herds. While all animals were RBT and CFT negative on the vaccination day, one cow resulted positive in the CFT but still negative in RBT about three months later (March 16th, 2010) (the E.U. eradication program allows using the CFT without RBT screening), and was culled. A bacteriological study of this cow conducted by the Official Veterinary Services gave negative results for field brucellae. Eight months later (November 18th, 2010), all cows remained negative in RBT and CFT and were revaccinated with a full dose of RB51 and kept isolated in the same farm. However, after two months (January 24th, 2011), 18 cows resulted positive in the CFT but RBT negative. The CFT was repeated twice by the official laboratory with positive results. On March 31st, 2011, those 18 cows were retested and found again CFT positive, and were slaughtered. In the subsequent bacteriological study conducted by the Official Veterinary Services, the cephalic, mammary, and iliac lymph nodes, plus the uterus, mammary gland, and spleen, were cultured on Farrell's selective medium (which inhibits RB51 but not field *B. abortus* strains; see Background) with negative results. Consistent with this negative search for field brucellae, the remaining cows in the herd were RBT and CFT negative 4 and 11 months later (May 30th and December 13th, 2011). Consequently, the affected farmer filed a lawsuit against the Official Veterinary Services and, based on a review of the serological, bacteriological, epidemiological, and clinical evidence by independent experts, the Justice Court concluded that the herd had never been affected by brucellosis, that the CFT positive results were caused by RB51 vaccination and revaccination, and sentenced the Official Veterinary Services to compensate the farmer [81].

Although consistently observed in this careful follow-up, simultaneous RBT negative and CFT positive results are unexpected because the antibodies against the R-LPS elicited by RB51 are not detected in RBT (or other agglutination tests), and both CFT and RBT use suspensions of whole S bacteria as antigens. However, the bacteria in these suspensions are heat-inactivated [82], a procedure that releases S-LPS exposing a proportion of the inner epitopes of the S-LPS and R-LPS molecules remaining in the outer membrane, and RBT and CFT work on different principles. Therefore, a plausible explanation for the negative RBT results is that the proportion of exposed core-lipid A epitopes is insufficient to make significant the binding of the same immunoglobulin molecule to different cells, the phenomenon causing visible agglutination. On the other hand, binding a single IgG molecule can trigger activation and subsequent amplification of the complement cascade, making CFT more sensitive to anti-R-LPS antibodies.

While testing RBT-negative animals by CFT is not a common strategy, these findings cast doubts on the usefulness of CFT to asses the significance of anamnestic responses detected by RBT in RB51 vaccinated animals that are in contact with field strains (see above).

### RB51 safety

As a general rule, mass vaccination is required to control bovine brucellosis when between-herd prevalence is high (the overwhelming problem in endemic countries), making vaccination of pregnant animals inevitable [25, 28, 83]. Nevertheless, depending on their degree of attenuation and the physiological status of the host, live *Brucella* vaccines convey safety risks intrinsic to the tissue tropism of these pathogens; namely, genital infections, abortions when applied to pregnant cows, and milk excretion. Hence, brucellosis vaccines/vaccination procedures should combine safety and protective efficacy, an issue mostly solved for S19 [25].

#### Abortions and premature deliveries

An initial observation in RB51 research was that intravenous inoculation of full doses during the sixth month of pregnancy causes placentitis and infection of the placentomes and uterus [84], showing that RB51 retains genital tropism. Subsequently, some works with limited numbers of animals concluded that pregnant cattle could be vaccinated and revaccinated subcutaneously with RB51 (including those previously immunized with S19) without inducing abortions. However, these studies used 1/10 of the full dose [5 heifers] [85], the intramuscular route [7 heifers] and an atypical vaccination procedure precluding practical conclusions [86], or are flawed by revaccinating S19 immunized animals (*n* = 57) in the 7th month of pregnancy [49, 50], too late for vaccine-induced abortions to occur [25]. Although the abortifacient effect of RB51 had already been shown (see below), more recent studies also attempted to address this question. In one, adult cows (*n* = 8) were inoculated with the full dose at 2 months of gestation and challenged with *B. abortus* 2308 between 6 and 7 months of pregnancy [61]. Although the authors did not observe abortions before the challenge, they did not test animals inoculated at mid-pregnancy, the correct time to assess this risk [25], and the bacteriological searches conducted in the challenged animals that aborted used Farrell’s medium, which as stressed above is highly inhibitory for RB51 (see Background). A study investigating RB51 innocuity at mid-pregnancy [87, 88] recorded 2 losses in 16 vaccinated animals, and because this figure was similar to that of the control [3 abortions in 16 animals], the authors assumed that RB51 was not a cause of abortions. Nevertheless, they did not provide the bacteriological evidence necessary to support their conclusion.

The evidence obtained through field studies and veterinary practice may also be valuable depending on several requisites. In line with works claiming the safety of RB51 under experimental conditions, two field studies reported no or meager rates of side effects irrespective of the pregnancy status at vaccination. In the Azores islands, 180,000 adult cattle were vaccinated with the full dose of RB51, and “*no side effects, such as abortions were recorded (passive reporting)*” (sic.) [89]. Passive reporting uses the voluntary declarations of farmers but because of the stigma and inconveniences of the official intervention that comes with the identification of infected farmsteads, owners are commonly reluctant to declare the existence of this problem [90, 91]. Thus, passive reporting is unreliable and even disadvantageous to follow these untoward effects in brucellosis. Similarly inconclusive is the evidence obtained in the bacteriological examinations (including 298 abortions) conducted in Azores: although RB51 was only isolated in one weak newborn calf, the medium used (Farrell’s) is highly inhibitory for RB51 (see Background). In Extremadura (Spain), a program based on mass vaccination with RB51 (full dose) with yearly revaccination with RB51 (up to four times) was applied to extensively bred cattle [92]. Following the vaccination of approximately 14,900 pregnant cows, 897 abortions (i.e., about 6%) were declared by farmers, and RB51 was cultured in 78 cases (fetuses, placentas, or vaginal swabs). Nevertheless, like in the Azores study, recording the abortions relied only on the willingness of farmers to declare they had a problem, and moreover 78 isolations is a surprisingly high number considering the very limited efficacy of the bacteriological procedure (Farrell’s medium) to detect RB51 (see Background). A case/control study in Portugal also claimed that RB51 was innocuous in pregnant cattle [93] (see also below). However, the authors did not describe the number of pregnant cows vaccinated, the pregnancy status and the critically important reporting methodology. The authors noted a significant decrease in birth rates after vaccination/revaccination, and, although they claimed that RB51 was not involved, the bacteriological follow-up used the RB51-inhibitory Farrell’s medium (see Background) and was also inadequate in other aspects. It is worth noting that the already existing and continuous recommendations of the OIE [2, 94] and the instructions of the manufacturer providing RB51 in the Azores and Extremadura cases [95] were ignored. Similarly disregarded were other studies and observations that had already returned negative results on RB51 safety a few years after the authorization in the U.S.A. in 1996.

**Fig. 3:**
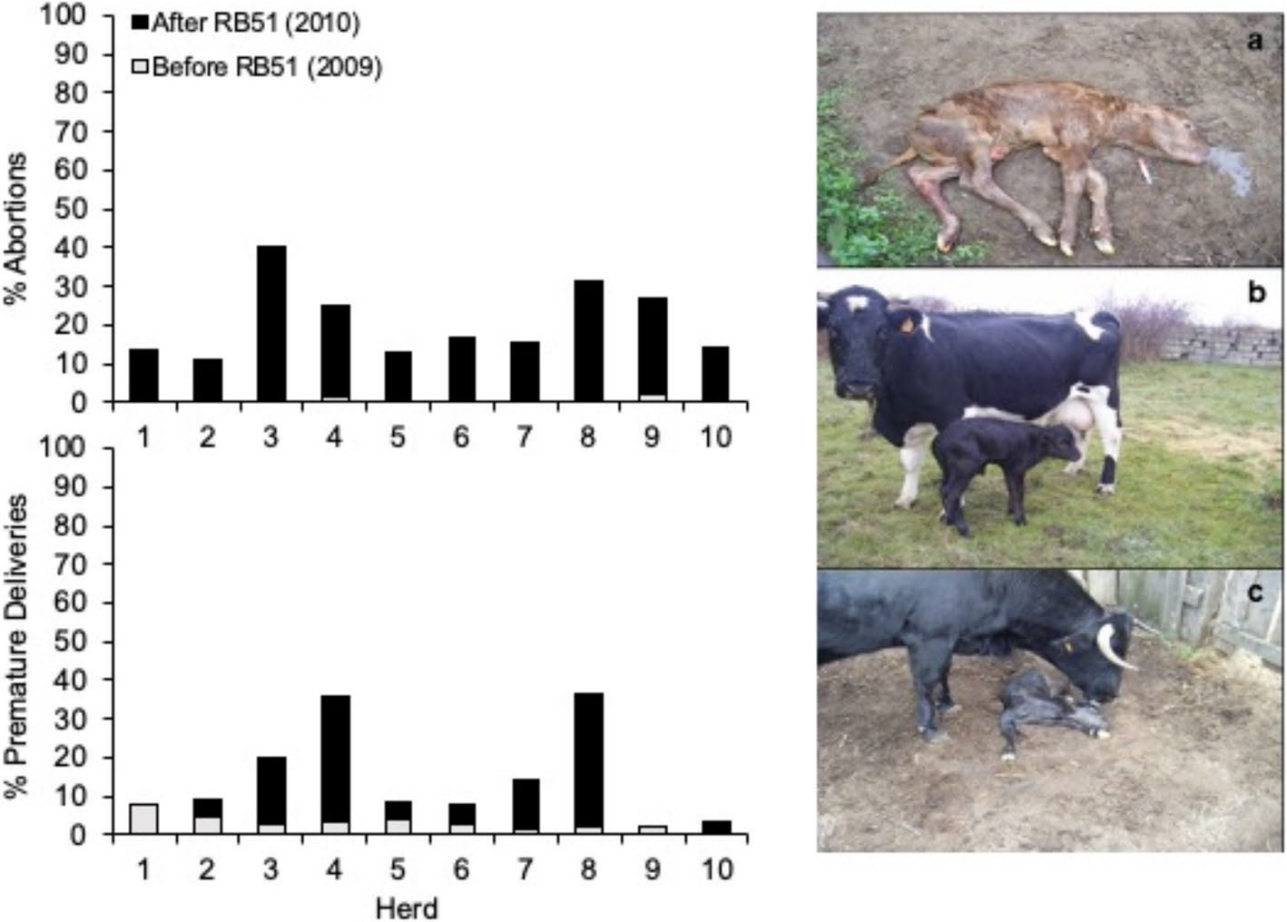
Abortions and premature deliveries after RB51 mass vaccination in brucellosis free herds in several Spanish regions. Left panels: abortions and premature deliveries in ten brucellosis free herds before (in white [year 2009]) and after (in black [year 2010]) subcutaneous vaccination and revaccination after six months with full doses of RB51 irrespective of the age and reproductive condition. RB51 was isolated from the abortions, vaginal swabs and milk of affected cows in herds 1, 4, 5, 7, 8 and 9 (herds 2, 3, 6 and 10 were not tested bacteriologically) (adapted from [101]). Right panel: abortions (**a**), weak newborns (**b**) and dead-born calves (**c**) commonly observed in brucellosis free herds submitted to whole vaccination (years 2009 and 2010). RB51 was isolated from the aborted material, vaginal swabs and/or milk samples (pictures kindly provided by O. García)

In 1998, Korea started a RB51 vaccination program. However, this program was immediately discontinued because of the “*unexpected*” (sic.; considering the claims disseminated up to this date) rates of abortion and premature births [96]. Likewise, the RB51 mass-vaccination trials conducted in Chile over 20 years ago were associated with frequent vaccine-induced abortions, and RB51 was isolated from high numbers of aborted cattle and cows at the moment of parturition [97]. Also, shortly after its authorization, the full dose of RB51 applied to a pregnant heifer was reported to cause necrotizing placentitis and endometritis with abortion, and the identity of the strain was confirmed by isolation and PCR identification [98]. Similar documented evidence was provided for 2 abortions of dairy cows investigated in Iran [99]. Two herds of pregnant adult cattle vaccinated with the full dose of RB51 in Wyoming in 2006 recorded 19 out of 360 and 3 out of 475 reproductive losses (abortions, stillbirths, premature calves, and unbred cows [presumed to be early abortions]), and the involvement of RB51 was confirmed by PCR. This event was a tenfold historical increment in reproductive losses for the herd, a damage over the limit tolerable by owners [100]. Like in Chile [97], those RB51-induced abortions were first noticed after three and half months, and a slightly shorter interval was reported in a large study in Spain [101]. Fifty-seven beef herds with 3346 cows, including 2249 (i.e., 67%) pregnant ones, were mass-vaccinated with the full dose of RB51. Subsequently, 47 herds reported 1.5% to 40% abortions and 2.3% to 50% premature deliveries, and 563 pregnant cows aborted or calved prematurely, in most cases between 60 and 90 days after vaccination. The mean of abortions and/or premature deliveries per herd was around 25%, in close agreement with previous observational studies in Spain [102]. The mean age of aborted fetuses was 7—8.5 months, with many dead-born or weak newborn calves. For 10 herds, there were records of abortions and premature deliveries in the year previous to vaccination (Fig. 3, left panels) and, like in the Wyoming case, the comparison showed a striking increase in adverse effects after vaccination with RB51. All animals were negative in standard brucellosis tests, proving that S-type brucellae were not involved. Also, vaginal discharges after abortion and milk from 6 of these herds were cultured on both Farrell’s and CITA (respectively inhibiting and allowing RB51 growth; see Background). Whereas bacteriology did not yield S brucellae on either medium, RB51 was consistently isolated on CITA agar, and identity confirmed by conventional and Bruce-ladder PCRs. Abortions, weak newborns and dead-born calves (illustrated in Fig. 3, right panel) were frequent in brucellosis-free herds submitted to whole herd vaccination with RB51 in Spain.

Other negative experiences concern the vaccination/revaccination protocols. Table 1 summarizes unpublished results from another area in Spain. The first set of observations (Table 1A) includes 840 animals distributed in 8 brucellosis-free herds where reproductive data were recorded regularly. All animals were mass-vaccinated and revaccinated six months later with full doses of RB51. As can be seen, the rate of abortions was very high, between 10 and 48%, and RB51 but no wildtype *B. abortus* was demonstrated in clinical specimens cultured using a suitable CITA’s and Farrell’s media combination (see Background). Throughout the study, all herds were serologically negative and no field brucellae were isolated. Most abortions took place 40–60 days after vaccination or revaccination, in some cases after 90 or more days, and the mean age of aborted fetuses and prematurely delivered calves was 7—8.5 months (Fig. 3 right panel). This evidence is further substantiated by observations made in an endemic area covering 13 municipalities [90 herds, 5600 animals, 3742 of which were pregnant] of unknown brucellosis sanitary status where the animals were vaccinated following the same protocol (Table 1B). Significantly, the rate of abortions and premature deliveries ranged from 12 to 40%, which does not differ from that recorded in the 8 brucellosis-free herds. Therefore, the conclusion is that the RB51-induced side effects are general because they were observed irrespectively of the brucellosis sanitary status of the herds. These untoward effects are relevant because the situation in mass-vaccination programs would be similar to that of those 90 herds. The efficacy of these mass-vaccination protocols regarding eradication is discussed below (*The experience of large-scale programs in cattle*).

**Table 1:** Abortions and premature deliveries after whole herd vaccination and revaccination with RB51 in Segovia (Spain)^a^

**A. In brucellosis free herds from which RB51 was isolated**^**b**^		
**Herds**^**c**^	**Abortions and premature deliveries/no. of cows (%)**	
	**in the herd**	**in pregnant**
A	1/41 (2.4)	1/10 (10)
B	5/50 (10)	5/20 (25)
C	9/110 (8.1)	9/50 (18.0)
D	16/150 (10.6)	16/100 (16.0)
E	17/135 (12.6)	17/110 (15.4)
F	19/122 (15.6)	19/95 (20.0)
G	29/126 (23)	29/60 (48.3)
H	22/70 (31.4)	22/58 (37.9)
TOTAL	804/118 (14.7)	503/118 (23.4)
**B**. **In herds of unknown individual brucellosis sanitary status at vaccination not submitted to bacteriological analyses**		
**Municipalities**^**c, d**^	**Abortions and premature deliveries/no. of cows (%)**	
	**in the municipality**	**in pregnant**
I	24/217 (11)	24/136 (17.6)
J	85/516 (16.5)	85/381 (22.3)
K	29/202 (14.3)	29/292 (21.4)
K	50/292 (17.1)	50/205 (24.3)
L	94/534 (17.6)	94/367 (25.6)
M	32/150 (21.3)	79/150 (40.5)
N	53/301 (17.6)	53/197 (26.9)
O	42/ 370 (11.3)	42/205 (20.5)
P	26/291 (8.9)	26/209 (12.4)
Q	63/480 (13.1)	63/270 (23.3)
R	106/1108 (9.6)	106/679 (15.6)
S	105/586 (17.9)	105/441 (23.8)
T	126/553 (22.8)	126/438 (28.7)
TOTAL	835/5600 (14.9)	835/3742 (22.3)

^**a**^In 2009 and 2010, crossbred cows of extensively reared herd were inoculated and then re-inoculated 6 months later with full subcutaneous doses of RB51 irrespective of the age and reproductive condition. Reproductive data were recorded regularly in all herds

^**b**^Representative samples (vaginal swabs and milk) from cows suffering abortion/premature delivery were cultured on both CITA’s and Farrell’s media and the isolates identified as RB51 by standard procedures and Bruce-ladder PCR

^**c**^Herds/municipalities identification replaced by capital letters

^**d**^Altogether, 90 herds

#### Vaccination of bulls with RB51

Genital tropism is a potential issue of live vaccines that also affects males and, based on two adverse reports [83], it is not advised to vaccinate bulls with S19 [2, 25]. For RB51, an initial study did not observe side effects after intramuscular administration of a full dose of RB51 to 6 sexually mature bulls [86]. Nevertheless, the study's non-representative route of inoculation and use of the RB51 inhibitory Farrell’s medium (see Background) impede conclusions. On the other hand, genital colonization and semen excretion of RB51 have been reported in 1 out of 6 bulls subcutaneously vaccinated with the full dose of RB51 [103]. Manufacturers recommend to use it only in female cattle [53, 104–106] and some explicitely indicate not to vaccinate males [95].

#### Human infections by RB51

Any shedding of live vaccines against zoonotic bacteria should always be considered from the perspective of human health risks. Regarding RB51, the first concern comes from its release by pregnant vaccinated animals aborting or calving prematurely (see above) or, in some animals without such symptoms, in the vaginal fluids [98, 107, 108]. Veterinary, medical professionals and animal handlers are risk groups in brucellosis [21] and, not surprisingly, occupational exposure gave humans the first proof of RB51 virulence. Shortly after its introduction in Chile, a veterinarian was unequivocally diagnosed with brucellosis by RB51 [109] and since then more cases affecting veterinarians, assistants, and students have been recorded in the U.S.A. [110, 111]. Although initially it was stated that RB51 is safer for practitioners than S19 [44], conclusive evidence for this is meager, and those U.S.A. reports may be an underestimation because the disease lacks pathognomonic symtoms [28] and serological tests for the diagnosis of human brucellosis do not detect exposure or infection by RB51 [112, 113]. Moreover, the U.S.A. CDC records may not reveal the extent of infections caused by accidental needle injuries among veterinarians [110]. Therefore, like other live brucellosis vaccines, RB51 strictly requires using personal protective equipment for vaccination and awareness of the risks when tending RB51 vaccinated animals [25]. These precautions must be extended to clinical laboratories testing suspicious samples [114].

A second source of problems results from the misconception that RB51 is safe for use in adult cattle and not excreted in milk, a conclusion of studies flawed by the use of Farrell’s medium [115, 116]. Others assumed that RB51 was not infectious for humans when unwisely stressing that, rather than a problem, RB51 excretion is a possible advantage because it creates a continuous oral immunostimulatory effect in the herd [50]. Indeed, like S19, RB51 is unlikely to colonize the mammary gland and be excreted in milk when used exclusively in 3–5 months-old calves. However, a common tendency is to vaccinate replacement animals later (at 8–14 months of age), which may include young pregnant heifers, and mass-vaccination includes pregnant cows. These practices multiply the risks because vaccination of adult cattle with RB51 can result in mammary gland/lymph node colonization and subsequent milk excretion of the vaccine in a significant number of animals [50, 84, 85, 101, 102, 117]. Such excretion is not an anecdotal event because it was reported to occur in close to half (5/13) of the adult vaccinated cows and can last for over two months after vaccination [50] or even continuously [78]. In the U.S.A., where over 75% of the States allow the marketing of unpasteurized milk [112, 118, 119] and about four million heifers are vaccinated yearly with RB51 [46], RB51 has been isolated from milk [120–122]. The hazard for humans is revealed by the number of cases repeatedly detected in this country [112, 120, 122, 123]. The persistence of RB51 in cheese has been shown under experimental conditions [124], and at least one human case has been traced to the consumption of unpasteurized cheese [125].

These problems are multiplied by the same antigenic characteristics that made this vaccine a potential alternative to S19. Human infections by RB51 cannot be diagnosed using the serological methods that detect anti-O-PS antibodies and are of routine use in clinical laboratories, and no RB51-specific antibody assay has been validated [112]. Considering the importance of these tests, the risks of misdiagnosis are evident, and the implications of a diagnostic failure are multiplied by the resistance of RB51 to rifampin [110, 112, 125]. This antibiotic is one of the first-line drugs for treating human brucellosis and other diseases [35]. Not surprisingly, to limit RB51 use, the United States Animal Health Association recommended in 2018 that state animal health officials and the cattle industry evaluate the need for RB51 vaccination in brucellosis free areas where *B. abortus* in wildlife is not a documented risk, like in the Great Yellowstone Area [47].

### RB51 protective efficacy

Valid information on the protection provided by cattle brucellosis vaccines is obtained through controlled experiments and field observations, which should be supported by further practical experience (i.e., performance in large-scale programs).

#### Controlled experiments in cattle

These experiments are a first step to unambiguously assessing vaccine performance and level of protection, even though they are expensive and technically demanding [25, 28, 30]. They are necessary because observations under the conditions of routine use are easily biased by several confounding factors, including the impact of complementary measures, which are always very important to control and eradicate this disease [21, 30]. However, controlled experiments are scientifically valid only under particular and not easy-to-implement experimental conditions [28]. These are: (i) sufficient numbers of brucellosis-free cattle of similar age, breed and physiological condition; (ii) challenge performed with an internationally recognized strain of virulence confirmed for absence of S to R dissociation and laboratory-caused attenuation, displaying the pertinent multiplication profile in mouse spleens [126] and used following the master-seed, seed-lot strategy; (iii) a route of challenge in accordance to internationally recognized methods, applied at mid pregnancy (when animals are most susceptible); (iv) a dose infecting a high proportion of unvaccinated controls (if a low proportion is infected, poor vaccines may show statistically significant efficacy); (v) a reliable bacteriological methodology (including selective media inhibiting contaminants but not the challenge or the vaccine strains) that yields unequivocal results (including the differentiation of challenge and vaccine colonies); (vi) an optimized sensitivity of detection; i.e., tissue homogenates of a complete set of organs and lymph nodes seeded in a suitable amount of each sample (and not merely tissue slices or minute amounts of dilutions from very small tissue samples) on at least duplicate culture plates. Indeed, there is room for variations within these guidelines. Therefore, it is necessary to examine critically the experimental protocols in published works, particularly when comparing different experiments because the many biological parameters involved are not uniform across different works [127]. This is why it is always advisable for vaccine evaluation to simultaneously include a control group vaccinated with S19 because reproducing the results reported for the reference vaccine of proven efficacy will inform about the adequacy of the above-summarized conditions. Of course, this S19-vaccinated group is indispensable to answer the question of whether a new vaccine affords better, less, or similar protection.

Very few controlled studies have assessed the protection afforded by RB51, and not all included an S19-vaccinated group. Table 2 summarizes those that did not and their relevant experimental details. As can be seen, the protection was highly variable. Whereas in Experiment 3, using the reduced dose, 100% of the RB51 vaccinated became infected (i.e., no protection), in Experiment 2 (also with reduced dose), the infection was 0% (i.e., 100% protection) against a *B. abortus* 2308 challenge infecting 100% and 67%, respectively, of unvaccinated cows. According to the best results (i.e., Experiment 2), unvaccinated cows had a 29.7 times higher relative risk (RR) of being infected than RB51 vaccinated cows, and 96.6% of the attributable infections (AF) in the former could have been prevented by vaccination. However, these RR and AF values were much lower in Experiments 1 and 4 (full dose given orally and subcutaneously, respectively) and also inferior in Experiment 3 (full dose). Particularly striking are the opposite results obtained with the reduced dose (claimed to be the safest in adult cows [64]) in two experiments and that the 100% protection obtained in Experiment 2 with the reduced dose is not reproduced with the full dose in any other experiment. In addition to these issues, methodological variations (route and age at vaccination, vaccination-challenge intervals, and bacteriological methodology) also prevent making conclusions in these experiments.

**Table 2:** Controlled experiments that evaluate the protective efficacy of RB51 in comparison only with unvaccinated controls

Experiment (reference)	Vaccine	Dose	Nº bovines	Nº infected (%)^a^	Relative Risk (CI)^b^	Attributable fraction (%)^c^
1 [128]^d^	RB51	1.0 × 10^10^	10	2 (20.0)	4.0 (1.11–14.35)	75.0
	Saline		10	8 (80.0)		
2 [64]^e^	RB51	1.0–3.0 × 10^9^	15	0 (0.0)	29.7 (1.92–458.3)	96.6
	Saline		6	6 (100)		
3 [129]^f^	RB51	1.0 × 10^9^	4	4 (100)	0.66 (0.46–0.95)	NC^g^
	RB51	1.0 × 10^10^	26	12 (46.0)	1.44 (0.83–2.49)	30.0
	Saline		15	10 (67.0)		
4 [61]^h^	RB51	1.5 × 10^10^	20	7 (35.0)	2.46 (1.02–5.88)	59.3
	Saline		13	11 (84.6)		
5 [65]^i^	RB51	1.0 × 10^10^	28	24 (85.7)	1.08 (0.87–1.33)	7.69
	Saline		14	13 (92.8)		

^a^In all experiments, infection was defined by bacteriological isolation of the *B. abortus* 2308 challenge strain but the methodology was not always similarly thorough (compare footnotes d, e, f, h and i)

^b^Relative Risk (RR) is the ratio of the probability of infection in the unvaccinated (control) and vaccinated groups. CI, confidence interval

^c^Population attributable fraction (AF) estimates the proportion of infection cases that could be avoided by vaccination

^d^In Experiment 1, the samples cultured were limited to vaginal fluids and milk obtained on day 3 and 14 after parturition, and parotid, iliac and mammary lymph nodes obtained at necropsy two months after parturition but only in non-excretors; lungs and abomasal fluids of fetuses were also examined

^e^In Experiment 2, the results of two groups vaccinated with 1 × 10^9^ and 3 × 10^9^ CFU are pooled; a thorough bacteriological search was performed, including samples of milk, vaginal fluids, placenta, and, after necropsy, mammary gland, spleen, liver, and the most important lymph nodes

^f^In Experiment 3, the bacteriological search included only the animals that could be necropsied and observed for abortion

^g^NC, not calculable

^h^In Experiment 4, specimens of parotid, retropharyngeal, prescapular, supramammary, internal iliac, and bronchial lymph nodes, mammary gland, lung, spleen, liver, milk, vaginal swab and placentome were cultured

^i^In Experiment 5, challenge was performed 4, 5 and 6 years after vaccination (results are pooled) and specimens of lymph nodes (bronchial, hepatic, internal iliac, mandibular, mesenteric, parotid, prescapular, retropharyngeal, and suprammamary), lung, liver, spleen and caruncle or placentome, plus samples of milk and mammary tissue were cultured

Table 3 summarizes the results of the few experiments in which RB51 has been evaluated in parallel with S19. Again, it is worth noting that not all experimental protocols are equivalent, and that some do not fulfill the above-listed experimental conditions of controlled experiments. Nonetheless, whereas these circumstances prevent statistical comparisons of different experiments [127], they are valid within a given experiment because all results are affected by the same biases. Keeping this in mind, in the experiments conducted with *B. abortus* 2308 as the challenge strain, RB51 only conferred significant protection when the infection rate in the unvaccinated controls was 60% (Experiments 6 and 8). As underlined above (experimental condition iv), these results strongly suggest that RB51 is not a very effective vaccine, an interpretation supported by Experiment 7, in which *B. abortus* 2308 infected 100% of the unvaccinated controls and RB51 failed to confer significant protection, while S19 did. The protection conferred by RB51 against *B. abortus* 544 as the challenge strain (Experiment 9) was also lower than that provided by S19, confirming that S19 is more efficacious than RB51 under the most stringent conditions. Consistent with the results of all experiments in Table 3, the RR and AF values were always higher in the cows vaccinated with S19, no matter the challenge pressure and the challenge strain. In summary, these controlled experiments show that protection against *B. abortus* by RB51 is low to moderate and always lower than that obtained with S19.

**Table 3:** Controlled experiments in which RB51 protective efficacy (full dose) was evaluated in parallel with S19 (standard dose)^a^

Experiment (reference)	Vaccine	Age at vaccination (months)	Nº bovines	Nº infected (%)	P *vs* Control^b^	Relative Risk *vs* control (CI)^c^	Attributable fraction (%)^d^
6 [62, 71]^e^	RB51	3–10	29	3 (10.3)	*P* < 0.01	5.8 (1.87–17.94)	82.7
	S19	3–10	22	1 (4.5)	*P* < 0.01	13.2 (1.88–92.6)	92.4
	Control	-	20	12 (60)			
7 (SENASA data)^f^	RB51	9	24	20 (83.3)	n.s.^g^	1.2 (1.00–1.43)	16.6
	S19	9	29	18 (62)	*P* < 0.05	1.6 (1.21–2.14)	37.5
	Control	-	22	22 (100)			
8 (NADC data)^h^	RB51	3–10	87	20 (22)	*P* < 0.01	2.59 (1.65–4.06)	67.7
	S19	3–10	19	1 (5)	*P* < 0.01	11.31 (1.65–77.37)	91.9
	None	-	47	28 (60)		-	-
9 [130]^i^	RB51	16–18	10	5 (50)	*P* < 0.05	2 (1.07–3.71)	50.0
	S19	16–18	9	2 (22.2)	*P* < 0.01	4.5 (1.32–15.27)	77.7
	None	16–18	10	10 (100)			

^a^In all experiments, a midgestational challenge was carried out and infection defined by bacteriological isolation of the challenge strain (*B. abortus* 2308 or *B. abortus* 544 in [130]). Bacteriological searches after challenge included at least milk, vaginal fluids, placenta and, after necropsy, mammary gland, spleen, liver and the most important lymph nodes. When described, bacteriological procedures are summarized in the footnote of each experiment

^b^Chi-square test

^c^Relative Risk (RR) is the ratio of the probability of infection in the unvaccinated (control) and vaccinated groups; CI, confidence interval

^d^Population attributable fraction (AF) estimates the proportion of infection cases that could be avoided by vaccination

^e^Challenge administered conjunctively and, for bacteriological analyses, tissues were grinded and tenfold dilution inoculated in tryptone-broth and then plated on agar

^f^The SENASA (Servicio Nacional de Sanidad y Calidad Agroalimentaria, Argentina) data correspond to two experiments [131, 132]; the challenge was applied intramuscularly; bacteriology was performed on tryptone agar

^g^n.s., not significant

^h^Overall National Animal Disease Center (NADC) data on efficacy of brucellosis vaccines after experimental challenge (elaborated from [56])

^i^Tissue slices or homogenates plated on agar *Brucella* agar base plates

Despite its extended use for decades, the duration of the immunity provided by the full dose of RB51 in calves has been examined only recently. In a controlled experiment (Table 2, experiment 5; data are pooled for simplification) in which calves were vaccinated with the full dose of RB51 and challenged 4, 5, and 6 years later, the respective rates of infected/vaccinated animals were 5/7, 8/9 and 9/12, versus 11/14 in the unvaccinated controls [65]. Obviously, the protection was not significant no matter the year after vaccination, and this waning is consistent with the low to moderate efficacy observed at shorter post-vaccination intervals in other controlled experiments. Even though the authors suggested the administration of boosters by 4–5 years of age in endemic areas as a way “*to maintain high levels of protection*” (sic.), the data do not prove that protection was either high or sufficient in intervals shorter than 4 years, and the effect of such revaccination is similarly unknown.

#### Field observations in cattle

In field studies, a careful analysis is essential to dissect the effect of a vaccine because many variables and confounding factors occur and are generally not present in controlled experiences. These variables include: i) brucellosis sanitary status before vaccination (i.e., the prevalence figures), ii) previous vaccinations, iii) herd size, iv) breed, v) management (extensive or intensive, beef, milk, mixed), vi) criterion for selecting the animals and, vii) degree of implementation and type of complementary measures (control of animal movements, T/S policy, farmer compensations, etc.). The latter are always of paramount importance because they have a direct positive impact (see below *The experience of large-scale programs in cattle*). Therefore, it is necessary to include parallel controls similar to the herds of intervention.

A study in Venezuela [133] reported that a reduced dose (5 × 10^9^ CFU) of RB51 was more effective than S19 vaccination for protecting cattle, and this work is admitted as evidence for RB51 efficacy in recent reviews [59]. However, such a conclusion is contradicted by the experiments in Table 3 and unjustified if the study is carefully examined. The bovines belonged to two infected herds (one with 39% seroprevalence) that, before vaccination, were individually selected through negative serology. However, this is an incorrect method introducing a significant bias in the analysis and interpretation of the results [25]: obviously, any previous contact with the pathogen, whether it results in a positive serological test in the moment of sampling or not, randomly biases the immunological state of the animals. Surprisingly, none of the 285 animals vaccinated with RB51 (140 in the herd with 39% seroprevalence) became seropositive in the highly sensitive BPAT [27] during the follow-up period despite being kept mixed with infected cows in infected herds. Considering the above-summarized evidence on the lack of DIVA properties of RB51 in infected environments, this result indicates that the vaccinated animals were not exposed to field brucellae, invalidating any conclusion on RB51 efficacy.

Similar studies interpreted as proof of the efficacy of RB51 were carried out in Azores Islands (Portugal) [89] and Extremadura (Spain) [54, 92]. They differ from the Venezuelan study in that RB51 vaccination was complemented with T/S of seropositive animals and other sanitary measures. These two field studies did not used controls to ascertain the effect attributable to T/S and the complementary sanitary measures implemented, procedures that can eradicate brucellosis without vaccination [25, 90]. Other flaws of these studies have been underlined above (see RB51 safety).

Finally, a case/control study [93] reported that whole-herd RB51 vaccination plus T/S eradicated brucellosis in some farms in Portugal, while T/S alone did not. The authors assessed the evolution of the apparent seroprevalence in 10 “case” units (vaccination and T/S) of the same holding in comparison with 10 “control” units (T/S alone) of different holdings. However, considering the “case” as 10 different herds is a mistake because, as stated, they were a single holding with 10 production units and thus a single epidemiological unit. Another flaw of this study was that, whereas the breeding/management practices could be assumed (they were not described) to be homogeneous in the 10 “case” units of the same holding, it is highly improbable that they were similar and homogeneous throughout the 10 epidemiologically unrelated “control” herds (not described either) and thus a correct control. For these experiments to be valid, control animals should be placed and managed together with the case ones (i.e., in the same holding). To further prevent valid comparisons, four “control” herds were entirely depopulated and ceased activity during the study, and only one of the remaining 6 purportedly control herds was seropositive at the end of the study. Consequently, the only significant fact in this report is that, despite the reduction of prevalence, the control measures implemented in the RB51 vaccinated “case” units failed to eradicate the infection in the holding because at least one of the units remained infected during the last two years of the study. Finally, despite a sharp decrease in birth rates after vaccination/revaccination (a RB51 side effect -see section *RB51 Safety* above-), the authors concluded that the study proved the innocuousness of RB51. However, such innocuousness was not adequately investigated by recording breeding data correctly, using an active reporting system, and testing the pregnant vaccinated cows using appropriate bacteriological methods (see also above), problems that make this claim untenable.

#### The experience of large-scale programs in cattle

The experience obtained in large programs is the touchstone of the usefulness of a vaccine [83, 127]. No such experiences provide a reliable example that RB51 can be significant in eradicating bovine brucellosis, which contrasts with the success of programs using S19. In Chile, the disease remains in some regions after practically 30 years of combining RB51 with T/S [134], and the same is true for nations or production systems that use RB51 and for which there is reliable information [135, 136]. The E.U. declared Spain officially free of brucellosis in cattle in 2022 [137]. Following this, some experiences are worth commenting on.

**Fig. 4:**
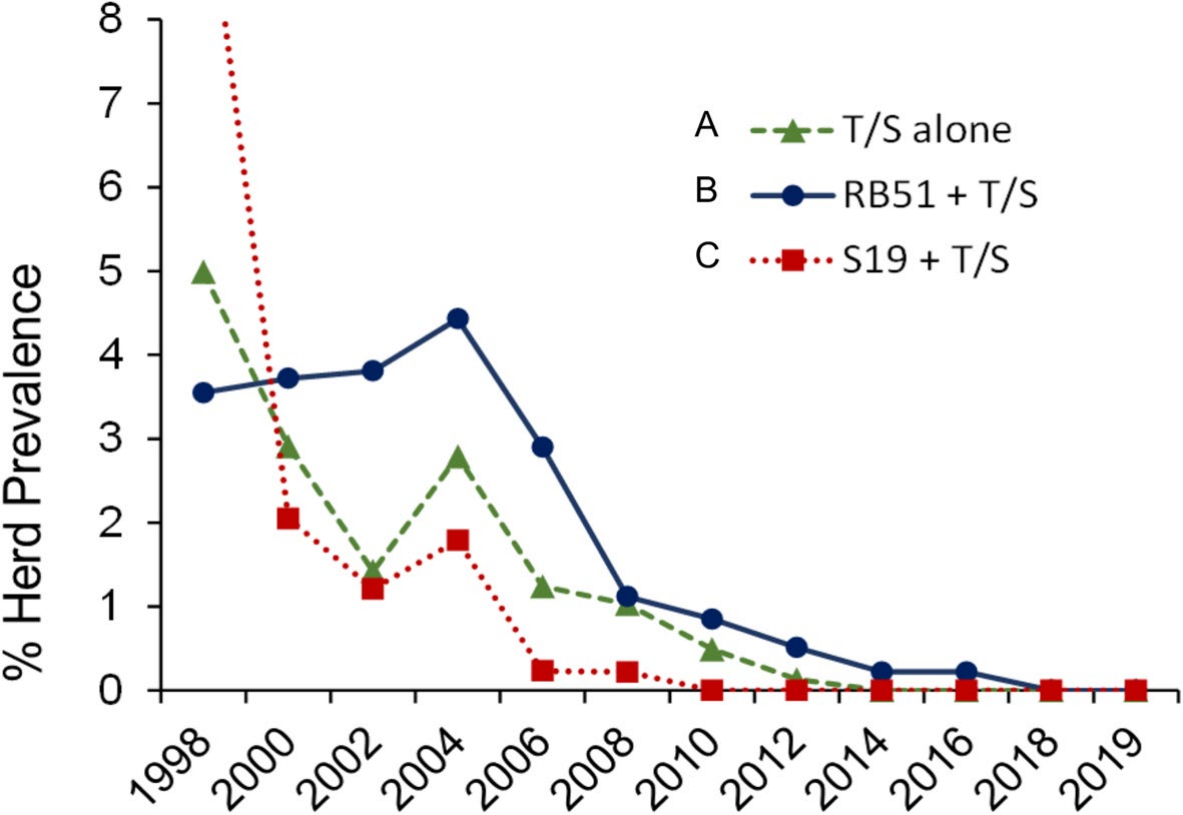
Efficacy of three eradication strategies applied in Spain. Until 1998, standard *B. abortus* S19 vaccination was applied in 3–6 months old calves throughout Spain, with variable intensity depending upon the region. Then, S19 vaccination was banned in all regions except Aragón, where coverage had been poor. After 1998 the strategies varied depending upon the regions. **A** T/S and no vaccination (European Union Directives 77/391/CEE and 78/52/CEE). Most regions attained eradication in 2012 and remained essentially free. The few outbreaks were cleaned by slaughtering the affected herds. **B** T/S and vaccination with RB51. Cantabria, Castilla-León, and Extremadura started the same T/S program as regions in A but herd prevalence increased progressively. In 2002, Cantabria started mass vaccination with full doses of RB51 plus revaccination 6 months later, irrespective of the age and reproductive condition, combined with TS (but see Safety section in the text), a strategey that became official also in Castilla-León and Extremadura in 2004. Altogether, 721,683 cows were vaccinated/revaccinated from 2002 to 2011 (no official data are available after 2011). Although prevalence decreased significantly, eradication was not achieved until 2018 (Castilla-León and Cantabria) or 2019 (Extremadura). **C** T/S and conjunctival vaccination with S19*.* Owing to the very high herd prevalence (over 16% in 1998, with about one third of herds infected by *B. melitensis*), Aragón implemented a compulsory S19 conjunctival vaccination program (5 × 10.^9^ CFU, either as one or two doses 2–3 months apart, or only a dose after year 2000) of 3–6 months old calves, combined with the European Union compulsory T/Sprogram. A total of 38,754 replacement calves were vaccinated from 2002 (no previous data are available) to 2010. Eradication was achieved in 2010, somewhat earlier than in the regions applying just T/S, and significantly faster than in those using RB51. Data from [138, 139]

Figure 4 shows the evolution of cattle brucellosis in several Spanish regions after 1998 according to official records [138, 139]. The measures applied to eradicate the disease were T/S (according to E.U. Directives) and RB51 or S19 vaccination in several combinations: (A) S19 vaccination banned after the year 1998 followed by only T/S (i.e., no vaccination); (B) S19 vaccination banned after the year 1998, followed first by T/S alone and, after 2004, by T/S plus RB51 vaccination and revaccination because of the increase in prevalence in the 1998–2004 period; and (C) T/S and S19 conjunctival vaccination of heifers. Both RB51 and S19 were provided by the same maker (CZ Vaccines, Porriño, Spain) and, where banned, S19 had been used as a full subcutaneous dose (see caption of Fig. 4 for other details). Several aspects are worth noting. First, starting from a relatively low prevalence, regions that used T/S and no vaccines (strategy A in Fig. 4) controlled and eventually eradicated cattle brucellosis in 2012 (the 2004 increase in prevalence is commented below). Second, for regions that followed strategy B (Fig. 4), T/S alone (1998 to 2004) failed to reduce the herd prevalence, which increased steadily. Third, after complementing T/S with RB51 vaccination/revaccination since 2004 in these regions, the progress was not faster than in the regions applying only T/S (in fact, eradication was achieved several years later), and caused significant side-effects (discussed above in section *RB51 safety*). Fourth, despite being very high in 1998, the herd prevalence dropped quickly in the region (Aragón) applying T/S plus conjunctival vaccination of replacement heifers with S19 (C in Fig. 4) and fell below those of other regions since 2002 until effective eradication in 2010.

Interestingly, a relatively high number of outbreaks took place in 2004 in the whole country, increasing the overall prevalence, but Aragón (the only region applying S19 at the time) had the lowest herd prevalence during these outbreaks. Also, eradication was attained somewhat earlier in Aragón than in regions that only used T/S and 8–9 years earlier than in regions with a much lower seroprevalence (in 1998) and that combined RB51 vaccination (from 2004 onwards) with T/S. It is also important to underline that in this region nearly one-third of the herds were infected by *B. melitensis* in 1998 (the prevalence of brucellosis in small ruminants was very high) and that the sporadic outbreaks after 2002 were always caused by *B. melitensis*. Altogether, these data question the alleged advantages of using RB51 when S19 is banned because it interferes with serological testing, the main reason for introducing R vaccines. On the other hand, the data prove the efficacy of S19 conjunctival vaccination against both *B. abortus* and *B. melitensis* infections of cattle and how, when correctly implemented, a program based on this vaccination strategy combined with T/S can control and achieve eradication more efficiently and faster than T/S, either alone or combined with RB51 vaccination. Noteworthy, T/S plus RB51 vaccination produced the worst results. While this may seem puzzling, it has been stressed before that immunization with a non-efficient vaccine creates a counter productive false sense of security [140].

#### RB51 protection of cattle against *B. melitensis*

As indicated above (Background) cattle can be permamently infected by *B. melitensis*, a common event in many resource poor countries. Therefore, a cattle vaccine should protect against infection by *B. melitensis.* In this regard, field experiences (like the above-discussed eradication in Aragón) confirm that S19 protects cattle against *B. melitensis* infections in cattle [6, 11], evidence lacking for RB51.

#### RB51 in other domestic animals and wild-life species

Controlled experiments show that RB51 is not efficacious against *B. abortus* infection of water buffaloes [141], does not protect sheep against *B. ovis* [142] or *B. melitensis* [143] or pigs against *B. suis* [144]. Because bison and wild cervids of the Great Yellowstone Area became infected through contact with cattle before *B. abortus* was eradicated in the latter, they may threaten the now brucellosis-free herds in neighboring areas. Since performance of S19 in bison is unsatisfactory [145], this experience led to investigate whether RB51 could protect these animals against *B. abortus.* In a study, 10 months old bison heifers were vaccinated with 4.5 × 10^10^ CFU of RB51 and challenged at midgestation with *B. abortus* 2308. All control animals (*n* = 8) were infected; among the 6 vaccinated, 3 were infected and 2 aborted [146]. This indicates that vaccination with RB51 is unlikely to be useful against *B. abortus* infections in bison. In experiments in captive elks, over 90% of the animals aborted after the challenge, no matter whether given the RB51 reduced dose, the full dose, or the full dose plus a booster [147, 148].

### Conclusion: RB51 delusions and facts

An ideal brucellosis vaccine should prevent infection with a single dose, be harmless, not transmitted to humans or other animals (including not to contaminate meat and edible organs, milk, and dairy products), be stable in vitro and in vivo*,* readily cultivable in large-scale, possess markers for an easy differentiation from field isolates, facilitate combined vaccination-T/S programs, and not stimulate antibodies interfering with serodiagnosis [149]. While not perfectly fulfilling all these ideal requisites, S19 has specific metabolic and molecular markers, its biological stability can be controlled in an in vivo test (the OIE mouse model), a single dose provides life-long good immunity, and applied by conjunctival route during calfhood lacks side effects and causes minimal serological interference. Indeed, programs combining S19 vaccination and T/S have been successful wherever cattle brucellosis has been eradicated (see above). Mass vaccination represents a different scenario in which abortions in pregnant cattle happen and the serological interference increases; both problems are considerably diminished when using the proper S19 dose and vaccination route [25]. Also, the relevance of the post-vaccinal serological interference depends mainly on whether T/S, with its high economic costs, repeated animal identification, and infrastructure demands, can be or will be effectively implemented. Regrettably, when mass vaccination is necessary, the answer to this question is usually negative [28].

There is evidence that repetition makes a fact seem true, regardless of whether it is or not, even if knowledge is available [150]. Concerning RB51, the propositions that it equals S19 in efficacy (i.e., protection, including mass vaccination, and proved role in eradication), meets the safety requirements, and is DIVA have become, through reiteration, post-truth misconceptions. A perusal of the literature shows that such properties are repetitively attributed to RB51 in reviews that, as shown here, do not examine critically the works cited, assume as accurate dated information, and/or do not include all evidence available [56, 59, 60, 151–154].

Strikingly, after three decades of use, there is no standard operating procedure for RB51, and different vaccination methods are used without solid proof of its effectivity and safety in all physiological states and epidemiological conditions possible. Concerning the protective efficacy, the perception that RB51 is a valuable vaccine owes some credit to the misconception that “protection against abortion” is an adequate index of vaccine efficacy, an error still found in recent meta-analyses and publications [64, 65, 153, 155]. Nevertheless, as forewarned many years ago [156], overlooking that some brucellosis vaccines can reduce clinical symptoms in a herd without clearing the infection is counterproductive because, if taken as an index of success, it gives a false sense of security and of progress that causes the perpetuation of the disease [140]. Indeed, it is known that vaccinated but infected cows that do not abort shed virulent brucellae copiously in vaginal fluids and milk and that a proportion of the calves born to such cows, although seronegative, are congenitally infected and will cause serious problems once they reach sexual maturity [24, 30, 127].

When scientific criteria are applied, several facts become clear. First, no controlled experiment supports that a single dose of RB51 matches S19, and all strongly suggest that it is only effective against moderate challenges. Second, the low protection provided by RB51 wanes in less than 4 years, possibly in much shorter intervals [65], and it is an unsupported assumption that revaccination with RB51 results in better protection. Third, these vaccination/revaccination protocols face important safety issues, practical inconveniences and increased costs in extensive breeding systems. Fourth, no valid study has ever proved that, when combined with complementary measures, including T/S, RB51 has been instrumental in eradication and that success (or progress) is not the result of such measures. On the contrary, the experiences of large programs discussed here strongly suggest that, under similar situations, such measures are at least as effective as a combined strategy that includes RB51 vaccination, whereas S19 combined with T/S allows a far more rapid control and eradication. Significantly, since the introduction of RB51 around three decades ago, no single country using this vaccine exclusively has controlled or eradicated bovine brucellosis.

Concerning safety, all solid evidence is against the idea that vaccination of pregnant cattle with RB51 has minimal side effects. Whereas this could be avoided in favorable circumstances, it is of the utmost importance to keep in mind that mass vaccination is the only strategy applicable under the conditions prevailing in most endemic countries or even in other situations when a large and prohibitive proportion of reactors would have to be removed [25, 56, 83]. Evidently, the pregnancy status of cattle is virtually impossible to ascertain when conducting whole-herd vaccination, particularly in resource-limited countries. The OIE warned against RB51 vaccination of pregnant animals in the 2004 edition of the Manual of Diagnostic Tests and Vaccines for Terrestrial Animals [94], a recommendation maintained until now [2]. Why the vaccine has been unwisely used in mass vaccination and revaccination, with the adverse effects documented here and the consequent harm to breeders, cannot be justified based on scientific facts.

Safety also encompasses the risk of human infections, either by professional exposure or consumption of raw milk and unpasteurized dairy products, both possibilities uncovered chiefly but not exclusively by the RB51 infections recorded in the U.S.A. The adverse effects of RB51 in humans include local erythema and induration, easily tracked to needle injuries when accidentally occurring during immunization, and the systemic manifestations of *Brucella* pathogenicity, including grave complications like neurobrucellosis [110, 125]. Since brucellosis lacks pathognomonic signs and symptoms, any suspicion must be confirmed by serological tests and/or bacteriological culture [157]. This requisite is a grave problem of this vaccine because RB51 infections are undetected in the agglutination tests regularly used as the first step in brucellosis diagnosis, making culture indispensable. However, culture requires specific equipment and repeated sampling before any antibiotherapy, and it has the delays, comparatively less sensitivity, and other difficulties intrinsic to *Brucella* detection and subsequent identification by this method [157]. These issues may not be critically important in countries like the U.S.A., where infections by RB51 can be suspected based on professional exposure or raw milk consumption and then confirmed by culture and an epidemiological investigation of easy to locate animals [108, 120, 122, 158]. However, most endemic countries have very limited possibilities of conducting *Brucella* cultures, and a negative serological test is interpreted as ruling out brucellosis, problems multiplied by the sad fact that in many endemic countries most dairy products are sold in informal markets without correct processing [159–162]. Thus, while representing a real risk, the extent of human infections associated with adult vaccination where RB51 is marketed is unknown because patients infected by RB51 are likely to be misdiagnosed or undiagnosed. From biosafety and ethical perspectives [163], the widespread authorization of an animal vaccine that is infectious for humans, not easily diagnosed and resistant to an antibiotic of choice to treat important ailments (including the disease it is supposed to combat) is perplexing. It is worth noting that none of these diagnostic and treatment problems is posed by S19.

Finally, the RB51 DIVA properties (the main reason for introducing this R vaccine) lack practical value. Because of contacts with field strains in endemic areas and the various degrees of accessibility of lipid A-core epitopes, serological tests cannot distinguish properly RB51 vaccinated not exposed, vaccinated exposed but not infected, and vaccinated infected individuals, issues accentuated by repeated RB51 vaccination or revaccination of animals previously immunized with S19. From a practical standpoint, any T/S policy after those RB51 vaccination practices would result in considerable over-culling because of the diagnostic confusion created. These facts nullify the alleged advantages of using RB51 and discontinuing or banning S19 because it interferes with serological testing. Unreasonably, RB51 has substituted S19 in countries that had never systematically applied (and still do not apply) T/S after vaccination, making diagnostic interferences irrelevant while safety, efficacy, and costs (RB51 is significantly more expensive than S19) are the pertinent criteria to select a bovine brucellosis vaccine [25, 28]. Also, its current use in the U.S.A. is striking.

In summary, RB51 DIVA, safety, and protection (and thus its overall efficacy) are post-truth delusions that have steadily grown since it was introduced, first in a country that had practically eradicated brucellosis and then in countries experiencing problems in the control of the disease due to multiple factors not related to S19 [28]. Nonetheless, both a proper understanding of the methodology of brucellosis vaccine research and the reality of the disease proves that the pieces on which these fabrications stand are incorrect and/or oversimplifications, revealing the critical deficiencies of RB51.

## Data Availability

Not applicable.

## Abbreviations

AF: Attributable fraction
BAPAT: Buffered acid plate agglutination test
ELISA: Enzyme-linked immunosorbent assay
iELISA: Indirect ELISA
cELISA: Competitive ELISA
CFT: Complement fixation test
CFU: Colony forming units
DIVA: Differentiation of infected and vaccinated animals
E.U.: European Union
FPA: And the fluorescence polarization assay
LFiC: Lateral flow immunochromatography
LPS: Lipopolysaccharide
O-PS: O-polysaccharide
R-LPS: Rough lipopolysaccharide
R: Rough
RBT: Rose bengal agglutination test
RR: Relative risk
S-LPS: Smooth lipopolysaccharide
S: Smooth
SAT: Standard agglutination test
T/S: Test-and-slaughter
